# Transcranial photoacoustic computed tomography based on a layered back-projection method

**DOI:** 10.1016/j.pacs.2020.100213

**Published:** 2020-10-16

**Authors:** Shuai Na, Xiaoyun Yuan, Li Lin, Julio Isla, David Garrett, Lihong V. Wang

**Affiliations:** a Caltech Optical Imaging Laboratory, Andrew and Peggy Cherng Department of Medical Engineering, California Institute of Technology, 1200 East California Boulevard, Pasadena, CA 91125, USA; b Caltech Optical Imaging Laboratory, Department of Electrical Engineering, California Institute of Technology, 1200 East California Boulevard, Pasadena, CA 91125, USA

**Keywords:** Photoacoustic computed tomography, Transcranial imaging, Back-projection, Reconstruction, Acoustic aberration

## Abstract

A major challenge of transcranial human brain photoacoustic computed tomography (PACT) is correcting for the acoustic aberration induced by the skull. Here, we present a modified universal back-projection (UBP) method, termed layered UBP (L-UBP), that can de-aberrate the transcranial PA signals by accommodating the skull heterogeneity into conventional UBP. In L-UBP, the acoustic medium is divided into multiple layers: the acoustic coupling fluid layer between the skull and detectors, the skull layer, and the brain tissue layer, which are assigned different acoustic properties. The transmission coefficients and wave conversion are considered at the fluid–skull and skull–tissue interfaces. Simulations of transcranial PACT using L-UBP were conducted to validate the method. *Ex vivo* experiments with a newly developed three-dimensional PACT system with 1-MHz center frequency demonstrated that L-UBP can substantially improve the image quality compared to conventional UBP.

## 1 Introduction

The brain is central to human characteristics and behaviors such as cognition, motion control, and language [1]. Spatial and temporal mapping of brain activities has shed light on how information are processed, has facilitated diagnosis and treatment of neurological disorders, and may advance technologies such as brain-machine communications [[2], [3], [4]]. Over the past few decades, magnetic resonance imaging (MRI) has been the workhorse for human brain imaging. However, MRI requires confining a subject in a noisy enclosure, has a non-linear relationship with paramagnetic deoxyhemoglobin (HbR), and suffers from substantial tissue background [[5], [6], [7]]. Moreover, the magnetic compatibility constraints, high cost and maintenance, and requirements of dedicated space limit its applications [8]. Single photon emission computed tomography (SPECT) and positron emission tomography (PET) are capable of visualizing metabolic process, but their use of radioactive isotopes make them unsuitable for many patient populations (e.g., children) or frequent use [9]. Electroencephalography (EEG), magnetoencephalography (MEG), and diffuse optical tomography (DOT) have relatively low costs and are free of radioactive isotopes, but suffer from low spatial resolutions [10,11]. Recently, functional ultrasound (fUS) has shown the capabilities of imaging human neonatal brain activities through the fontanelles [12]. Nevertheless, the two-way skull-induced acoustic aberration remains an obstacle for its translation to transcranial imaging of human adults.

Photoacoustic (PA) computed tomography (PACT) is an emerging technique that generates images noninvasively with optical contrasts at centimeter-scale depths. The PA signals are produced by irradiating tissues with a non-ionizing diffuse laser pulse. Light absorption launches thermoelastically induced ultrasonic waves, which are subsequently recorded by ultrasonic detectors and used to reconstruct the distribution of light absorption [13]. At certain near-infrared wavelengths, PA signals are almost exclusively from hemoglobin (Hb), which is orders of magnitude more absorptive than other tissue components. Intrinsically sensitive to optical absorption with a 100 % relative sensitivity, PACT can measure the concentrations of both oxyhemoglobin (HbO_2_) and HbR based on their distinct spectral signatures in a proportional relationship, allowing quantifications of both concentrations [14]. PACT systems can be transportable, open, and quiet, minimizing site requirements and patient/subject stress [15,16].

Despite recent advances, the major remaining challenge associated with human brain PACT is correcting for the skull-induced acoustic aberration, i.e., wave-front distortion and attenuation. To date, the most widely used PACT reconstruction algorithm is universal back-projection (UBP), which assumes an acoustically homogeneous medium [17]. However, this assumption is violated in transcranial PACT due to the presence of the skull. Several attempts towards transcranial PACT or thermoacoustic tomography (TAT) have been reported, where the images were either optimized by tuning the speed of sound (SOS) in a simplified homogeneous medium or reconstructed using a heterogeneous model, but without considering the wave conversion at the skull boundaries [[18], [19], [20], [21], [22]]. These approaches can enhance regional contrasts by compensating or correcting for the SOS heterogeneity but cannot suppress and sometimes may introduce artifacts due to the ignorance of wave conversion at the skull boundaries. More computationally intensive and complex methods based on the elastic wave equations have also been investigated [[23], [24], [25]]. Although these approaches can solve the wave propagation relatively accurately, the computational cost is generally high due to the requirements of solving the entire wave propagation, posing potential challenges in efficiently processing large image stacks. Consequently, there is a demand for fast but simple reconstruction algorithms that can correct for the dominant aberration factors in transcranial PACT.

In this work, we present a modified UBP algorithm, termed layered UBP (L-UBP), to correct for the skull-induced acoustic aberration. The algorithm is based on the prior knowledge of the skull geometry and its position relative to the ultrasonic detectors. The ultrasound propagation medium is divided into three layers: the homogenous acoustic coupling fluid layer between the ultrasonic detectors and the outer skull boundary, the skull layer, and the brain tissue layer inside the skull. In L-UBP, the acoustic refraction and wave conversion at the layer interfaces are considered, and the skull attenuation and transmission coefficients are compensated for. In Section 2, the principle and mathematical description of L-UBP are presented. In Section 3, simulations of L-UBP for transcranial imaging are performed to validate the method. Section 4 presents a three-dimensional (3D) PACT system developed to examine the proposed algorithm. Next, *ex vivo* transcranial imaging using the developed 3D PACT system is performed to demonstrate the proposed reconstruction method. Discussion and conclusion are provided in Section 5.

## 2 Principle of L-UBP

In the UBP algorithm, the initial pressure at ***r*** in an acoustically homogeneous medium can be recovered by

(1) $$p_{0}(r)=\frac{2}{\Omega_{0}}\int_{A_{0}}^{}\left(p\left(r^{'},t\right)-\frac{t\partial p\left(r^{'},t\right)}{\partial t}\right)d\Omega ,\;$$

where $p_{0}$ is the initial pressure of the PA source at r, $p(r^{'},\;t)$ denotes the pressure at $r^{'}$ and time $\;t=\left|r-r^{'}\right|/c$, c is the SOS in the medium, and $\Omega_{0}$ represents the solid angle of the whole detection surface $A_{0}$ with respect to the reconstruction point enclosed by $A_{0}$ [17]. In Eq. (1), $t\partial p(r^{'},\;t)/\partial t\gg p(r^{'},\;t)$ holds due to the high ultrasound frequency (>1 MHz) used for medical imaging. The derivative term $\partial p\left(r^{'},t\right)/\partial t$ is equivalent to a pure ramp filter in the frequency domain, which suppresses the low-frequency components of the PA signals. Since a realistic ultrasonic detector has a limited bandwidth, which functions similarly to the ramp filter in the frequency region below the center frequency, $\partial p\left(r^{'},t\right)/\partial t$ can be approximated as the measured pressure $p_{m}\left(r^{'},t\right).$ Therefore, Eq. (1) can be simplified to

(2) $$p_{0}\left(r\right)=-\frac{2}{{\text{Ω}}_{0}}\int_{{\text{A}}_{0}}^{}tp_{m}\left(r^{'},t\right)d\text{Ω}.$$

**Fig. 1:**
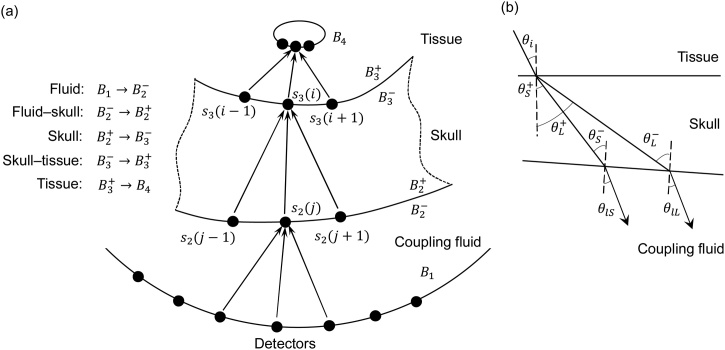
**Principle of** L-**UBP.** (**a**) Schematics of L-UBP. (**b**) Ultrasound transmission at simplified skull boundaries.

To account for the heterogeneity of the acoustic properties, the wave propagation medium is divided into three adjacent layers as shown in Fig. 1(a). They include the acoustic coupling fluid layer between the detectors and the outer skull boundary, the skull layer, and the tissue layer inside the skull, resulting in four boundaries: *B*_1_ to *B*_4_. Realistically, the acoustical properties within the skull layer are heterogeneous [25,26]. However, to allow for a fast algorithm, the skull layer is simplified as a single-layer structure with a homogeneous SOS and density, and only the primary aberration events occurring at the fluid–skull and skull–tissue interfaces are accounted for [22]. On the inner and outer skull boundaries, we define reconstruction grids with a size of λ/4, where λ is the ultrasound wavelength corresponding to the upper cut-off frequency. The PA signals recorded by the detectors are back-projected to the nodes on the outer skull boundary using conventional UBP. To compute the wave propagation successively, the computed pressure signals on the outer skull boundary are subsequently back-projected to the nodes on the inner skull boundary using conventional UBP. The same procedure is repeated to reconstruct the objects inside the skull from the nodes on the inner skull boundary.

**Fig. 2:**
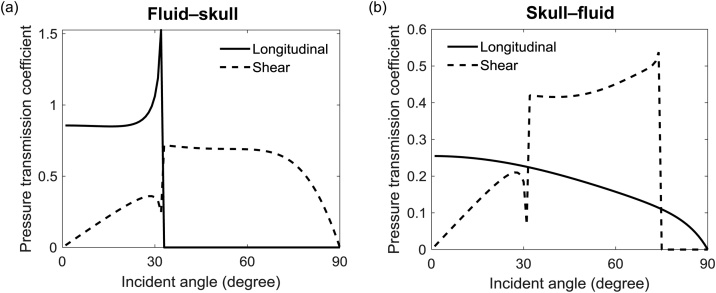
**Pressure transmission coefficients at the skull boundaries in water.** (**a**) Pressure transmission coefficients versus incident angles at the fluid–skull interface and (**b**) the skull–fluid interface.

It is important to note the wave conversion at the tissue–skull and skull–fluid interfaces since the bone supports both longitudinal and shear waves while the tissue and acoustic coupling fluid primarily support longitudinal waves. Fig. 1(b) illustrates the forward PA wave transmission at the skull boundaries which are simplified as two flat surfaces. When a PA longitudinal wave approaches the tissue–skull interface at incident angle $\theta_{i}$, it is partially refracted at exit angle $\theta_{L}$ and partially converted to a shear wave at exit angle $\theta_{S}$. The longitudinal and shear waves inside the skull convert back to longitudinal waves when leaving the skull, and propagate into the acoustic coupling fluid. Given the energy loss during the reflection and propagation of the reflected waves within the skull layer, and for simplicity and computational efficiency, wave reflections within the skull are not considered [22,26]. Since the tissue and acoustic coupling medium (normally water) have similar acoustic properties, they are both treated as fluid in the following discussion. Based on the theory of elastic and the boundary conditions, the pressure transmission coefficients at the fluid–skull interface can be derived as

(3) $$T_{fs\_L}=\left(\frac{\rho_{f}}{\rho_{s}}\right)\frac{2Z_{L}\text{cos}\left(2\theta_{S}^{+}\right)}{Z_{L}{\text{cos}}^{2}\left(2\theta_{S}^{+}\right)+Z_{S}{\text{sin}}^{2}\left(2\theta_{S}^{+}\right)+Z_{l}}\;,$$

(4) $$T_{fs\_S}=-\left(\frac{\rho_{f}}{\rho_{s}}\right)\frac{2Z_{S}\text{sin}\left(2\theta_{S}^{+}\right)}{Z_{L}{\text{cos}}^{2}\left(2\theta_{S}^{+}\right)+Z_{S}{\text{sin}}^{2}\left(2\theta_{S}^{+}\right)+Z_{l}}\;,$$

(5) $$Z_{L}=\frac{\rho_{s}c_{L}}{\text{cos}\theta_{L}^{+}}\;,\;Z_{S}=\frac{\rho_{s}c_{S}}{\text{cos}\theta_{S}^{+}}\;,\;Z_{l}=\frac{\rho_{f}c_{f}}{\text{cos}\theta_{i}}\;,$$

where subscripts f and s respectively represent the fluid and skull, $Z_{L}$ and $Z_{S}$ denote the acoustic impedance of the skull for longitudinal and shear waves divided by the cosine of their exit angles, and $Z_{f}$ denotes the acoustic impedance of the incident longitudinal wave in the acoustic coupling fluid divided by the cosine of the incident angle [27]. $\rho_{s}$ and $\rho_{f}$ indicate the density of the skull and acoustic coupling fluid, respectively. $c_{f}$, $c_{L}$, and $c_{S}$ represent the SOS of the longitudinal waves in the coupling fluid, the longitudinal waves in the skull, and the shear waves in the skull, respectively. Based on the reciprocity theory of wave propagation [28], the pressure transmission coefficients at the skull–fluid interface are

(6) $$T_{sf\_L}=\left(\frac{\rho_{f}}{\rho_{s}}\right)\frac{2Z_{f}\text{c}\text{o}\text{s}(2\theta_{S}^{-})}{Z_{L}{\text{cos}}^{2}\left(2\theta_{S}^{-}\right)+Z_{S}{\text{sin}}^{2}\left(2\theta_{S}^{-}\right)+Z_{fL}}$$

(7) $$T_{sf\_S}=-\left(\frac{\rho_{f}}{\rho_{s}}\right)\frac{2Z_{f}\text{sin}\left(2\theta_{S}^{-}\right)}{Z_{L}{\text{cos}}^{2}\left(2\theta_{S}^{-}\right)+Z_{S}{\text{sin}}^{2}\left(2\theta_{S}^{-}\right)+Z_{fS}}\;,$$

(8) $$Z_{L}=\frac{\rho_{s}c_{L}}{\text{cos}\theta_{L}^{-}}\;,\;Z_{S}=\frac{\rho_{s}c_{S}}{\text{cos}\theta_{S}^{-}}\;,\;Z_{fL}=\frac{\rho_{f}c_{f}}{\text{cos}\theta_{fL}}\;,\;{\;Z}_{lS}=\frac{\rho_{f}c_{f}}{\text{cos}\theta_{fS}}\;,$$

where $\theta_{fL}$ and $\theta_{fS}$ denote the exit angles of the longitudinal and shear waves transmitted from the skull into the coupling fluid. Based on Eqs. (3), (4), (5), (6), (7), (8), the pressure transmission coefficients of the longitudinal and shear waves versus their incident angles at the fluid–skull and skull–fluid interfaces are plotted in Fig. 2. The computation adopted representative acoustic property values $\rho_{l}=1000\;\text{k}\text{g}/{\text{m}}^{3},\;\rho_{s}=1800\;\text{k}\text{g}/{\text{m}}^{3},\;c_{L}=2800\;\text{m}/\text{s},c_{S}=1444\;\text{m}/\text{s},\;{\text{a}\text{n}\text{d}\;c}_{f}=1500\;\text{m}/\text{s}$ [22]. Fig. 2(a) shows that, at the fluid–skull interface, the critical angle for an incident longitudinal wave is approximately 33 degrees, below which the incident wave will be partially converted to both longitudinal and shear waves. Above the critical angle, only shear waves can be converted and transmitted into the skull layer. Fig. 2(a) shows that the pressure transmission coefficient can be greater than unity due to the higher acoustic impedance of the longitudinal wave in skull than that in water although the intensity transmittance can never surpass unity. Due to the higher SOS of the longitudinal waves in the skull layer than that in water, Fig. 2(b) shows no critical angle for the longitudinal wave at the skull–fluid interface but shows the critical angle for the shear wave around 75 degrees. In L-UBP, the longitudinal and shear waves undergo individual back-projection and are merged to form the final reconstructed image inside the skull. Since water does not support the propagation of shear waves, the longitudinal and shear waves in the skull are mixed before being detected. L-UBP therefore requires two back-projections of the total detected signals, which may be considered a limitation compared with the full-wave-based inverse solvers. However, considering that L-UBP is developed to reduce computational cost while enhancing blood vessel signals at the imaging site, one can further employ advanced full-wave-based algorithms to improve the reconstruction.

In L-UBP, the pressure on the outer skull boundary $B_{2}^{-}$ is first computed using conventional UBP. The transmission coefficients in Eqs. (3) and (4) are then used to calculate the pressure on the inner skull boundary $B_{2}^{+}$. The longitudinal and shear waves are then propagated through the skull to $B_{3}^{+}$, which can be computed using

(9) $$p\left(i,t\right)=-\frac{2}{\Omega_{0}}\sum_{j=1}^{N}\Delta t\left(i,j\right)p\left(j,t+\Delta t\left(i,j\right)\right)\Omega \left(i,j\right)\gamma \left(T,\varepsilon ,i,j\right)\;.\;$$

In the above equation, $\Delta t\left(i,j\right)$ represents the time of flight between the *j*-th node on $B_{2}$ (denoted by $s_{2}(j)$) and the *i*-th node on $B_{3}$ (denoted by $s_{3}(i)$), N is the total number of nodes on $B_{2}$, and $\Omega \left(i,j\right)$ denotes the solid angle of the effective area of $s_{2}(j)$ with respect to $s_{3}(i)$. To compensate for the transmission loss on $B_{3}$ and the acoustic attenuation between $\;B_{2}^{+}$ and $B_{3}^{-}$, a compensation factor γ is introduced as a function of the transmission coefficients and acoustic attenuation factors

(10) γT, i, j,ε=β1Tsf_Li,jeαLωys3⃑i-s2⃑j,ε , longitudinal ,β1Tsf_Si,jeαSωys3⃑i-s2⃑j,ε ,  shear . 

In Eq. (10), $\alpha_{L}$ and $\alpha_{S}$ are the acoustic attenuation coefficients of the longitudinal and shear waves, ω is the angular frequency, y is the power law exponent, and β is a cut-off function in the form of

(11) $$\beta \left(x,\varepsilon \right)=\left\{\begin{array}{c} x,\;\left|x\right|<\;\varepsilon \;,\;\\ 0,\;\text{o}\text{t}\text{h}\text{e}\text{r}\text{w}\text{i}\text{s}\text{e}\;. \end{array}\right)$$

Eq. (11) is used to avoid overcompensation of the transmission and attenuation losses: A large γ between the PA source and ultrasonic detectors corresponds to a small transmission coefficient, which can result to a low signal-to-noise ratio (SNR) of the signals; If the low-SNR signal is over-compensated for, the image quality will be degraded. The rule of thumb is to set ε equal to or smaller than the SNR of the temporal signals reconstructed at the node. Meanwhile, to avoid interception between the back-projected rays and the skull boundaries in both the coupling fluid and skull layers, only those rays with ≤ 90° incident angles at the skull boundaries are used for reconstruction.

## 3 Simulation

**Fig. 3:**
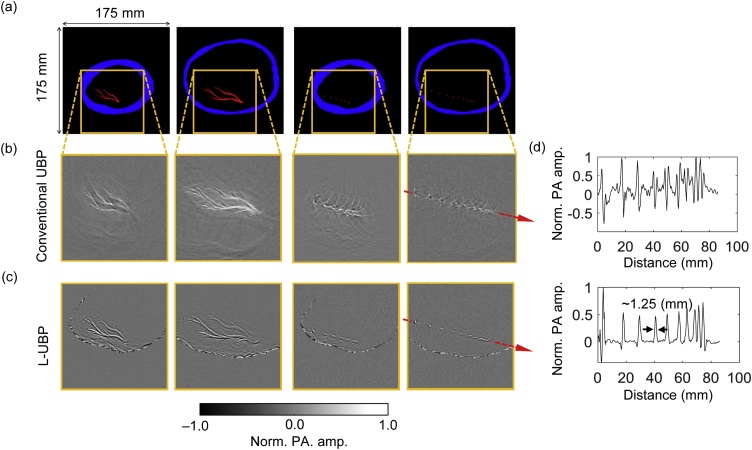
**2D simulations of transcranial PACT.** (**a**) Skull model with synthetic objects and point sources. (**b**) Objects and point sources reconstructed using conventional UBP. (**c**) Objects and point sources reconstructed using L-UBP. (**d**) Profiles of the aligned point sources in column 4. Norm., normalized, amp., amplitude.

Two-dimensional (2D) simulations were conducted to validate and compare L-UBP to conventional UBP. The simulations were conducted in 2D to reduce the computational cost but can be extended to 3D cases. As shown in Fig. 3, we extracted the outer and inner boundaries of an *ex vivo* adult human skull model from its 3D x-ray computed tomography (CT) data with a 0.3-mm isotropic resolution. The skull layer is defined by the blue regions in Fig. 3(a). The acoustic attenuation coefficients of the skull were set to be $\alpha_{L}=2.8\;\text{d}\text{B}/(\text{M}\text{H}{\text{z}}^{2}\text{c}\text{m})$ for the longitudinal wave and $\alpha_{S}=5.6\;\text{d}\text{B}/(\text{M}\text{H}{\text{z}}^{2}\text{c}\text{m})$ for the shear wave according to [22]. The black regions in Fig. 3(a) represent the coupling fluid (water) and tissue which are defined with the same attenuation coefficient $\alpha_{f}=0.1\;\text{d}\text{B}/(\text{M}\text{H}{\text{z}}^{2}\text{c}\text{m})$ and SOS $c_{f}=1500\;\text{m}/\text{s}$. Synthetic objects (presented in red in Fig. 3(a)) and aligned point sources are defined in the parietal (columns 1 and 3) and temporal (columns 2 and 4) regions of the skull. The simulations were performed in the open-source *K*-wave platform; an impulse signal was assigned to the object pixels at t=0 s to represent the initial PA pressure [29]. The forward propagation of the acoustic waves was simulated in the time domain and recorded at the detector positions enclosing the skull. The detectors were configured with a center frequency of 1 MHz and one-way -6–dB fractional bandwidth (FBW) of 78 %. The detection aperture consists of 600 elements evenly distributed on a circumference of 225-mm radius. The images reconstructed by both conventional UBP and L-UBP methods are displayed in Fig. 3(b)–(c). For conventional UBP, the SOS has been optimized by maximizing the average PA amplitude across all point source locations. Nevertheless, the reconstructed images demonstrate strong aberration due to the SOS inhomogeneity and ignorance of the wave conversion at the interfaces. Fig. 3(c) shows the reconstructed images using L-UBP and exhibits a better representation of the targets shown in Fig. 3(a). Moreover, the profiles of the aligned point sources reconstructed using L-UBP demonstrate a nearly isotropic resolution of ∼ 1.25 mm, defined by the full width at half maximum (FWHM). The results obtained using conventional UBP show highly distorted point spread functions (PSFs). In contrast to Fig. 3(b) and (c) also displays the inner skull boundary. This phenomenon is induced by the infinite solid angle of a pixel with respect to the node (virtual detector) on the boundary that overlaps the pixel. In reality, to avoid instability (zero denominator), we artificially assigned a small distance between the node and its overlapping pixel when computing the solid angle, resulting in large reconstructed pixel amplitudes (bipolar) on the inner skull boundary.

## 4 *Ex vivo* results

**Fig. 4:**
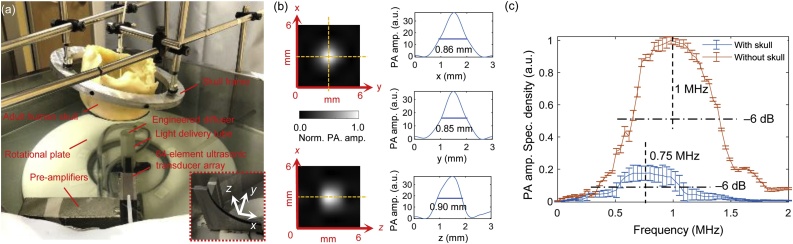
**3D PACT system.** (**a**) The 3D PACT system and an *ex vivo* adult human skull put in the system’s field of view (FOV). (**b**) The spatial resolution quantified by the FWHM of a reconstructed PA point source placed at the center of the system in homogeneous water (left column) and their profiles along three orthogonal directions (right column). (**c**) PA amplitude spectral densities measured using a PA point source placed at the center of the system with and without the skull. Error bars represent standard deviations across *n* = 64 × 600 detector positions distributed on the scanned hemispherical surface. Amp., amplitude, spec., spectral.

We constructed a 64-element 3D PACT system to perform *ex vivo* transcranial imaging (Fig. 4(a)). The system consists of four main modules: a laser module to excite PA signals, a 64-element quarter-ring detector array to record the PA signals, a 64-channel one-to-one-mapped signal amplification and acquisition devices to amplify and digitize the detected signals, and a scanning mechanism to rotate the detector array for azimuthal sampling over a hemisphere. The laser module employs a Q-switched Nd: YAG laser (Quanta-Ray PRO-350-23 10, Newport Spectra-Physics, Ltd.) emitting light pulses of 1064-nm wavelength with 8–12-ns pulse width. The pulse repetition frequency (PRF) and maximum pulse energy are 10 Hz and 2.2 J, respectively. The 64-element quarter-ring detector array (Imasonic, Inc.) was designed with a diameter of 20 cm, element size of 3 × 3 mm^2^ with a center frequency of ∼1 MHz and one-way – 6-dB fractional bandwidth of ∼78 %. All detector elements are evenly distributed along the quarter ring and directly connected to one-to-one mapped 40-dB preamplifiers. The amplified signals are digitized by a 64-channel 14-bit data acquisition (DAQ) system (Octopus Express 8389 CompuScope, DynamicSignals, LLC) with the sampling rate and amplification configured to 10 MHz and 14 dB, respectively. The quarter-ring array is driven by a step motor below the rotational plate to evenly sample 600 positions over 360 degrees to form a hemispherical detection aperture. Deionized water is used as the acoustic coupling medium.

We imaged a point absorber at the center of the system in water to quantify the spatial resolution. As shown in Fig. 4(b), the measured spatial resolution in the *x*-*y* plane is ∼0.85 mm, and the measured spatial resolution in the *z* direction is ∼0.9 mm. The relatively lower *z* resolution results from the fewer detectors along the latitude direction of the synthesized hemispherical aperture. Water has a high optical absorption coefficient at 1064 nm (0.1 cm^–1^), so to improve the illumination efficiency, a light tube was used to reduce the light travel distance in water between the emission aperture and the skull. An engineered diffuser (EDC-50, RPC, Inc.) mounted at the top of the light tube is used to homogenize and expand the laser beams to ∼5 cm in diameter on the skull. The resulting radiant exposure and fluence rate are about 40 mJ/cm^2^ and 400 mW/cm^2^ on the skull surface, both within the ANSI safety limit [30]. Fig. 4(c) shows the PA amplitude spectral densities with and without the skull present averaged across all detection positions on the hemispherical aperture. It demonstrates that the transcranial signals are shifted down in frequency compared to the signals recorded without skull. This shift is mainly attributable to the frequency-dependent attenuation that suppresses the high frequency components. Additionally, the peak PA amplitude spectral density was decreased by ∼ 83 % due to the presence of the skull.

**Fig. 5:**
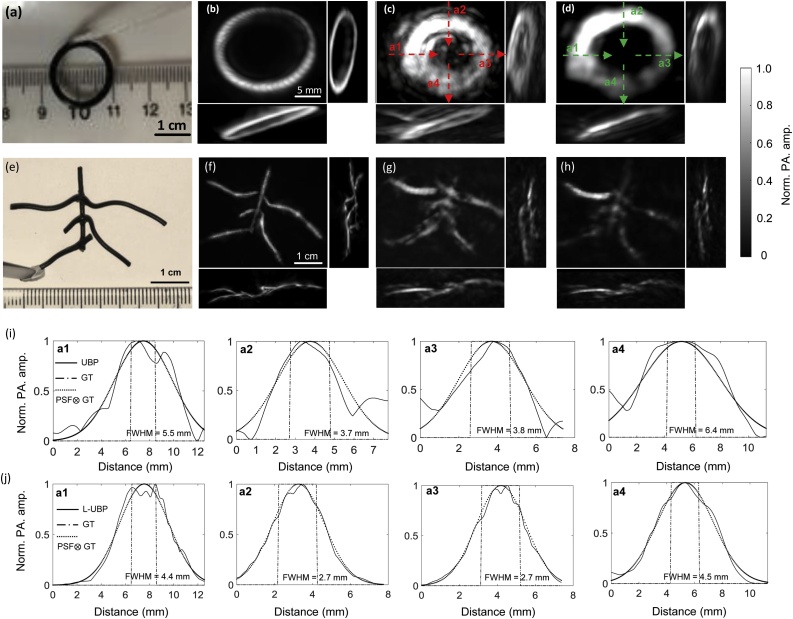
***Ex vivo* transcranial PACT results.** (**a**) Photograph of a PA absorber made of a black rubber O-ring. Images reconstructed using conventional UBP from data acquired (**b**) without and **(c**) with the skull present. (**d**) Image of the O-ring reconstructed using L-UBP. (**e**) Photograph of a PA absorber made of black electrical wires. (**f–h**) The images of the wires were reconstructed using the algorithms in the same orders with (b) to (d). All PACT images are presented in the form of maximum amplitude projection (MAP), and pixel values below 5 % of the maximum were thresholded to enhance visualization. 3D rendering can be found in Supplementary Movie 1. (**i**) Image profiles along the dashed arrow lines at four locations a1 to a4 in (c). (j) Image profiles along the dashed arrow lines at four locations a1 to a4 in (d). Norm., normalized, amp., amplitude.

The same 3D x-ray CT image of the adult human skull used in the simulation study was used to infer the skull boundaries. Since the Nyquist FOV of the current system configuration (assuming 1.2-MHz (<–6 dB) ultrasound cutoff frequency for the transcranial signals (Fig. 4(c)) and 600 scanning steps) is about 12 cm in diameter, the skull was only partially immersed to ensure all defined nodes were enclosed by the FOV. The skull was registered to the scanner’s coordinate system using four fiducial features, which could also be identified in the CT image. The positions of the fiducial features relative to the ultrasonic detector array were measured at four rotational angles spaced at 90 degrees. The skull model was fitted into the scanner’s coordinate system based on rigid transformation using the coordinates of the features in the CT space. Two PA absorbers, one made of a black rubber O-ring (Fig. 5(a)) and the other made of thin rubber wires (Fig. 5(f)), were placed at the skull temporal region ∼5 mm away from the inner boundaries. The two objects were imaged separately without and with the skull present. Fig. 5(b, f) and (c, g) display the images of the objects reconstructed using conventional UBP without and with the skull present, respectively. Compared to the reconstructed image without the skull in Figs. 5(b, f) and (c, g) reveal strong skull-induced distortions. In comparison, the distortion is suppressed in the L-UBP reconstruction shown in Fig. 5(d and h) demonstrating the effectiveness of the de-aberration algorithm. In Fig. 5(i) and (j), we quantified the transcranial resolutions of conventional UBP and L-UBP using the images in Fig. 5(c) and (d). Since the resolution is apparently location-dependent due to the acoustic aberration, four evenly distributed regions of interest (a1 to a4 along the arrow directions) were used for analysis. We assumed the image PSF as a zero-mean normal Gaussian function with a varying standard deviation (STD). By convolving the ground truth (GT) dimension of the PA source (indicated by the dashdotted lines in Fig. 5(i) and (j)) with the PSF of different STDs, we determined the best match between the convolution results (dotted lines) and the measured profiles (solid lines) through cross correlation. Then the STD corresponding to the largest correlation coefficient was used to define the PSF and compute the FWHM (shown at the bottom of each subfigures). Overall, the analysis at the four locations demonstrates an average transcranial resolution of ∼4.9 ± 1.3 mm for the conventional UBP, and an average resolution of ∼3.6 ± 1.0 mm for L-UBP. Although L-UBP has shown significant improvement over conventional UBP, the image reconstructed by L-UBP still have lower quality than those in Fig. 5(b and f). The degraded image quality mainly results from four factors. First, as shown in Fig. 4(c), the spectrum of transcranial PA signals is shifted to lower frequencies resulting in a low spatial resolution. Although the high frequency components have been compensated for using the frequency-dependent correction factor in Eq. (10), they could not be fully corrected for due to the degraded SNR of these high-frequency components. Second, the skull layer is simplified from an acoustically heterogeneous medium to a homogeneous one, inducing model inaccuracy. Third, co-registration between the skull and PACT system may introduce errors to the skull model, which is due to uncertainty in measuring the skull position relative to the lab coordinates using skull fiducial features. Fourth, the skull was previously stored in the air environment which caused air to be trapped in the skull dipole layer. Although the skull was vacuumed in a water chamber before experiments, the small residual amount of air trapped in the dipole layers might have worsen the homogeneity of the skull model. In addition, non-uniform pixel value distributions can be observed across the images. This is mainly due to the non-uniform illumination on the objects because of the non-uniform light transmission at the local skull regions.

**Fig. 6:**
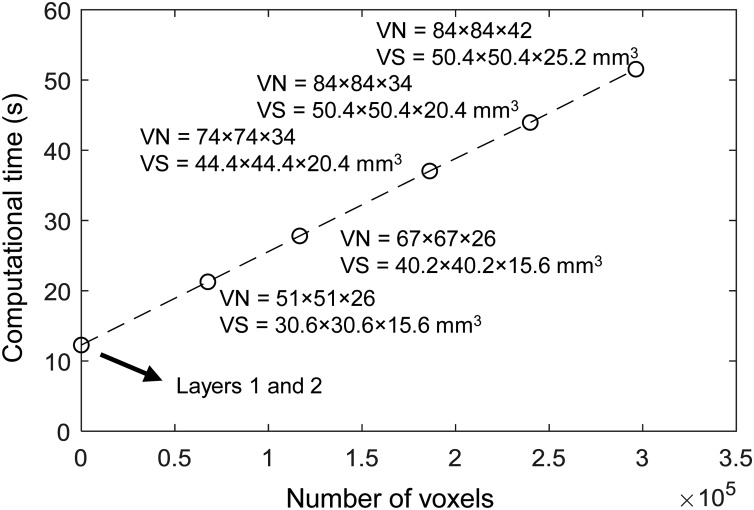
**Computational time versus number of voxels.** VN., voxel number, VS., volume size.

Fig. 6 shows the computational time versus number of voxels for 3D L-UBP. The speed test was performed on a personal computer equipped with CPU Intel i7-6800 K and GPU NVIDIA GeForce GTX-1080Ti (3584 CUDA cores). The same scanning parameters (transducer element count and azimuthal scanning steps) used in the above *ex vivo* experiment were employed. The reconstructed volume size with the shown voxel numbers and 0.6 × 0.6 × 0.6 mm^3^ voxel size are given. Since all voxels are defined inside the final layer, the computational time spent in the final layer increases linearly with the number of voxels. The processing time spent in the first two layers is independent of the number of voxels.

## 5 Conclusion and discussion

In this work, a de-aberration reconstruction methodology for transcranial PACT was proposed. The method, termed L-UBP, is based on conventional UBP and considers wave refraction and conversion at the boundaries between skull and surrounding media. Computer simulations were conducted to validate this method. A 3D imaging system was designed and constructed to investigate the effectiveness of the proposed method using an *ex vivo* adult human skull. Although L-UBP simplifies the skull model as a single layer structure with homogeneous acoustic properties, the transcranial image quality has shown substantial improvements compared to conventional UBP. Since the proposed method does not introduce extensive computational cost or GPU memory requirements, it can potentially be employed for batch processing of *in vivo* functional image stacks.

As shown in Fig. 4(a), the current system illuminates the skull in free space from the bottom. We have found that such illumination strategy is most efficient and introduces minimum complexity to the hardware. Nevertheless, free-space illumination can cause interference between hair and the excitation light, which requires the subject to shave hair (if any). Alternatively, fiber bundles attached to the scalp can be used for illumination, but will cause ∼ 50 % laser energy loss and may interfere with the acoustic field (if they are placed in the detection path). The current system requires the subject to be imaged in a prone position in order to partially immerse his/her head in water. To overcome this limitation, one can redesign the scanner for sitting-position imaging by inverting the current design. In an inverted design, acoustic coupling water can potentially be held by use of an acoustically and optically transparent film with external negative pressure to maintain the chamber pressure.

Another practical challenge associated with implementing L-UBP *in vivo* is to model the skull noninvasively. Here, we propose two roadmaps—subject-specific and subject-nonspecific approaches, for further investigations. For the subject-specific approach, either x-ray CT or high-resolution MRI structural images, which are acquired using MP2RAGE and PETRA sequences, can be used to model the skull [31,32]. Although less preferred compared to MRI due to the radiation, CT can directly provide the skull density map in Hounsfield units, which can then be used to estimate the elastic modulus and SOS of the bone [33]. For MRI, the MP2RAGE sequence offers a high contrast-to-noise ratio (CNR) and resolution, and PETRA is an ultra-short echo (UTE) sequence ideal for bone imaging. The UTE MR image will also provide a converted pseudo Hounsfield scale (HU) map that can facilitate estimation of the geometric and acoustic parameters of the skull [34]. Moreover, L-UBP algorithm can be extended to include more layers when using the subject-specific skull model. Although a high-resolution image is always desirable, some applications can tolerate a lower resolution if the image can be generated quickly, such as stroke imaging. As a result, we note an alternative subject-nonspecific approach which may offer onsite visualization of the brain function without depending on MRI or x-ray CT. It can potentially be realized by using the inner scalp boundary reconstructed by the conventional UBP algorithm to represent the skull outer boundary. This estimation is not affected by the presence of the skull when half-time UBP is used because the first arrival of the ultrasonic signal does not propagate through the skull [35]. Next, the inner skull boundary can be estimated by use of an atlas model [36], where the average skull thickness distribution can be counted statistically based on established databases. In the subject-nonspecific approach, the acoustic properties of the skull will be assumed homogeneous and can be assigned with average values as suggested in [[21], [22], [23], [24]].

## Declaration of Competing Interest

L.V.W. has a financial interest in Microphotoacoustics, Inc., CalPACT, LLC, and Union Photoacoustic Technologies, Ltd., which, however, did not support this work. The other authors declare no competing interests.
